# First Hominoid from the Late Miocene of the Irrawaddy Formation (Myanmar)

**DOI:** 10.1371/journal.pone.0017065

**Published:** 2011-04-20

**Authors:** Jean-Jacques Jaeger, Aung Naing Soe, Olivier Chavasseau, Pauline Coster, Edouard-Georges Emonet, Franck Guy, Renaud Lebrun, Aye Maung, Aung Aung Khyaw, Hla Shwe, Soe Thura Tun, Kyaw Linn Oo, Mana Rugbumrung, Hervé Bocherens, Mouloud Benammi, Kamol Chaivanich, Paul Tafforeau, Yaowalak Chaimanee

**Affiliations:** 1 Institut International de Paléoprimatologie et de Paléontologie Humaine, UMR CNRS 6046, Université de Poitiers, Poitiers, France; 2 Department of Geology, Dagon University, Yangon, Myanmar; 3 Institut des Sciences de l'Evolution de Montpellier, Université Montpellier 2, Montpellier, France; 4 Department of Archaeology, National Museum and Library, Mandalay, Myanmar; 5 Myanmar Geosciences Society, Yangon, Myanmar; 6 Department of Geology, University of Yangon, Yangon, Myanmar; 7 Paleontology Section, Department of Mineral Resources, Bangkok, Thailand; 8 Universität Tübingen, Institut für Geowissenschaften, Biogeologie, Tübingen, Germany; 9 Bangkok, Thailand; 10 European Synchrotron Radiation Facility, Grenoble, France; University of Oxford, United Kingdom

## Abstract

For over a century, a Neogene fossil mammal fauna has been known in the Irrawaddy Formation in central Myanmar. Unfortunately, the lack of accurately located fossiliferous sites and the absence of hominoid fossils have impeded paleontological studies. Here we describe the first hominoid found in Myanmar together with a *Hipparion* (*s.l.*) associated mammal fauna from Irrawaddy Formation deposits dated between 10.4 and 8.8 Ma by biochronology and magnetostratigraphy. This hominoid documents a new species of *Khoratpithecus*, increasing thereby the Miocene diversity of southern Asian hominoids. The composition of the associated fauna as well as stable isotope data on *Hipparion* (*s.l.*) indicate that it inhabited an evergreen forest in a C3-plant environment. Our results enlighten that late Miocene hominoids were more regionally diversified than other large mammals, pointing towards regionally-bounded evolution of the representatives of this group in Southeast Asia. The Irrawaddy Formation, with its extensive outcrops and long temporal range, has a great potential for improving our knowledge of hominoid evolution in Asia.

## Introduction

### Paleontological research in the Irrawaddy Formation and recent findings

Despite more than a century of intense research on the issue, the origin of the Pleistocene to extant orang-utan *Pongo* is poorly understood. Some Asian Miocene genera - namely *Ankarapithecus* (Turkey), *Sivapithecus* (India, Pakistan, Nepal), *Indopithecus* (India, Pakistan), *Lufengpithecus* (Yunnan province, China) and *Khoratpithecus* (Thailand) - have been linked to the *Pongo* clade [1], [2]. *Khoratpithecus*, the most recently discovered member of this clade, was interpreted as the closest fossil relative of *Pongo* [3], [4], suggesting that continental Southeast Asia was an important centre of the Miocene radiation of the *Pongo* clade. The long temporal range and extensive outcrops of the Irrawaddy Formation in Central Myanmar, where sediments range from the middle Miocene to the Pleistocene [5], [6], offer unique opportunities to complete the Asian hominoid fossil record. The Irrawaddy Formation has yielded numerous terrestrial mammal remains since the 19^th^ century [7]–[12]. However, despite these numerous reports, only a few precisely located fossiliferous sites have been described from this Formation [13], affecting the reliability of most of paleontological studies. This situation is well illustrated by the Yenangyaung region, type area of the Irrawaddy Formation, whose fossils were interpreted as belonging either to a single fauna [14] or to two faunas [10], [15], [16], and variously dated [6].

**Figure 1:**
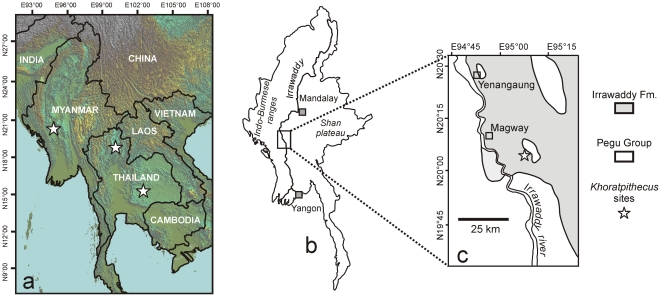
Location of the *Khoratpithecus*-bearing localities of Thailand and Myanmar. a: map of Southeast Asia. b: general map of Myanmar. c: local map of the region of Magway displaying the Irrawaddy Formation outcrops [6] in light grey.

First in 2002 and then yearly since 2006, the Myanmar-French paleontological mission has surveyed the Irrawaddy Formation outcrops of Central Myanmar with the aim of improving the comprehension of mammalian evolution in the Miocene of Southeast Asia. The present study describes a new mammal assemblage including hominoid remains collected in Irrawaddy Formation outcrops situated south of Magway city (Figure 1), during the 2006 and 2007 field seasons.

### Geological setting

Tertiary to Quaternary Irrawaddy fluvial deposits are widely exposed throughout the central basin of Myanmar. The continental deposits of the Irrawaddy Formation can reach thickness of 2000 m and span a time interval from Middle Miocene to Pleistocene [6].

Our field research area covers around 650 square kilometers from the north of Yenangyaung to the South of Magway, where hundred meters thick sections are accessible. In that area, the deposits of the Irrawaddy Formation comprise yellow to brownish coarse cross-bedded sandstones with interstratified ferruginous conglomerates, claystones, hardened fine sandstones and red soil horizons. It frequently contains iron hydroxide-rich nodular concretions and gypsum deposits. Lateral facies variation commonly occurs along the sections. The dipping of beds is low and rarely exceeds 10°. Minor angular and erosional unconformities have been observed within the sequence.

## Results and Discussion

### Systematic Paleontology Superfamily Hominoidea Gray 1825, Family Hominidae Gray 1825, Subfamily Ponginae Elliot 1913, Genus *Khoratpithecus* Chaimanee et al. 2004, *Khoratpithecus ayeyarwadyensis* sp. nov

urn:lsid:zoobank.org:act:B073A2A8-C53F-4D59-B7C8-06E50EBAB1E3

#### Holotype

Left hemi-mandible with P_3_-M_2_ (MFI-K171, collection of the Paleontology Section of the Department of Mineral Resources (DMR), Bangkok – 10400, Rama VI Road, Thailand).

#### Diagnosis

Species of *Khoratpithecus* differing from *K. piriyai* by a more slender corpus at nearly similar P_3_ to M_2_ size with low surface relief, much shorter symphysis with shorter planum alveolare, deeper genial fossa and more convex anterior border, narrower incisor area, smaller incisor alveoli with a greater proclination and a more pronounced narrowness, smaller M_3_ close in size to the M_2_, buccal walls of molars more slanted, smaller P_4_ with shorter and lower talonid and without individualized talonid cusps, more inclined alveoli of incisors, smaller male canine alveolus. Differs from *K. chiangmuanensis* by having molars with smaller central fovea, a P_3_ with a smaller distal basin, and a P_4_ with a smaller talonid basin and a lower mesiodistal-buccolingual ratio.

#### Comparative description of the holotype

**Figure 2:**
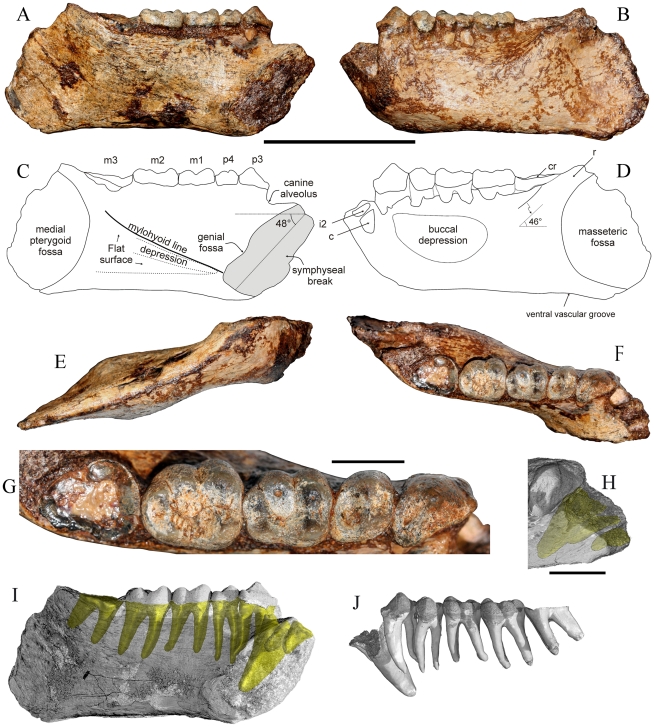
MFI-K171, holotype mandible of *Khoratpithecus ayeyarwadyensis* n. sp. A: lingual view. B: buccal view. C: interpretive drawing of the lingual view. D: interpretive drawing of the buccal view. E: ventral view. F: occlusal view. G: enlarged occlusal view of the tooth row. H: Detailed view of the anterior region displaying alveoli shadows. Scale bars: 5 cm for A–F and I–J and 1 cm for G–H. Abbreviations: cr, crown remnant; r, ramus. I: lingual view displaying roots shadows for P_3_-M_3_ and alveoli shadows for incisors and the canine. J: dental row with virtually extracted roots in buccal view.

MFI-K171 is a nearly complete and undistorted left hemi-mandible (Figure 2) discovered by a villager from Magway area, and retrieved by one of the authors (K. C.) who deposited it in the collections of the DMR (Bangkok). This mandible was associated with several mammalian taxa identical to those collected by our team. The specimen is broken anteriorly close to the midsagittal plane, allowing a good approximation of the symphyseal section. Both incisors and the canine are missing. Nevertheless, their alveoli are partially preserved. The corpus, coated by an iron hydroxide-rich matrix at the time of the discovery, shows no trace of deformation. The holotype exhibits perfectly preserved P_3_ to M_2_ crowns but most of the crown of the M_3_ was broken away. Enamel is nevertheless observable in the mesial, buccal and distal part of the M_3_ remnant, allowing the extrapolation of the measurements at the cervix. Access to the inner anatomy of the specimen was rendered possible by the use of X-ray microtomography techniques. Distally, MFI-K171 is interrupted 1 cm posterior to the trigonum retromolare. A part of the angular area of the corpus is preserved. The ramus is nearly entirely lacking.

MFI-K171 has an overall slender morphology owing to a shallow and thin corpus. It possesses a nearly constant depth from the distal edge of the symphysis to the M_3_, where it is only slightly greater (Table 1). MFI-K171 corpus is shallower than that of *K. piriyai*, TF 6223, both in terms of absolute depth and depth in relation to tooth size (Table 2). The thickness of the corpus of MFI-K171 is least at the level of the P_4_-M_1_ transition (Table 1) and increases moderately until the M_3_ where the lateral flaring is maximal. However, it is narrower than the corpus of TF 6223, in particular at the level of M3 where there lateral eminence of TF 6223 is much stronger (Table 2). Gracility indices of MFI-K71 and TF 6223 yield close values at P_4_ and M_1_ but differ markedly at M_3_ (Table 2). Comparison with 9 sexed mandibles of the Miocene hominoid *Ouranopithecus macedoniensis* (5 females, 4 males; data from [17] and own measurements), suggest that the difference in corpus thickness at M_3_ between TF 6223 and MFI-K171 is unlikely to represent intraspecific variation: the range of the corpus thickness vs. M_1_ size index is more than 5 times greater than that of the whole sample of *O. macedoniensis* (Table 2). The difference in gracility index between the two *Khoratpithecus* mandibles is similar to the total range of *Ouranopithecus* and greater than the intrasex *Ouranopithecus* ranges.

**Table 1:** Mandibular measurements of *Khoratpithecus ayeyarwadyensis* (in millimeters).

Mandibular measurement	Distance
Maximal length of the specimen	102.4
Corpus depth at P_3_	32.60/35.92 (buccal/lingual)
Corpus depth at P_4_	30.44/35.84 (buccal/lingual)
Corpus depth at P_4_-M_1_ transition	30.89/35.19 (buccal/lingual)
Corpus depth at M_1_ talonid	32.07 (buccal)
Corpus depth at mid M_2_	31.86 (buccal)
Corpus depth at mid M_3_	36.51 (buccal)
Corpus depth at the distal extremity of M_3_	39.59 (buccal)
Corpus thickness at P_4_	17.87
Corpus thickness at P_4_-M_1_ transition	15.95
Corpus thickness at mid M_1_	16.43
Corpus thickness at mid M_2_	18.67
Corpus thickness at M_3_	21.39
Corpus thickness at distal extremity	3.72
Length of the molar row	41.15
Length of the premolar row	19.23
P_3_-M_3_ length	61.02
I_1_ mesial margin-M_3_ distal margin length	70.56
Alveolar medio-external breadth of the incisors	8.86
Alveolar medio-external breadth of I_1_-C	14.37
Symphysis section long axis	34.81
Symphysis section short axis	17.33
Symphysis section height	29.62
Symphysis section length	30.13

**Table 2:** Comparison of the corpora measurements and indices of MFI-K171, TF 6223, and *Ouranopithecus macedoniensis*.

Level	MFI-K171	TF 6223	*O. macedoniensis*
**Corpus depth**			
P_4_	33.14	38.88	-
M_1_ trigonid	33.04	40.61	29.29–43.65
			29.29–31.89(n = 5)/41.67–43.65(n = 3)
Mid-M_3_	36.51	40.19	26.29–41.29
			26.29–30.48(n = 5)/37.88–41.28(n = 2)
**Corpus thickness**			
P_4_	17.87	21.23	-
M_1_ trigonid	15.98	19.84	13.10–20.18
			13.10–17.37(n = 5)/16.15–20.18(n = 4)
Mid-M_3_	21.39	31.43	20.52–26.32
			20.52–23.41(n = 5)/24.69–26.32(n = 4)
**Corpus depth vs. M_1_ size** a			
P_4_	2.89	3.30	-
M_1_ trigonid	2.88	3.46	2.28–3.38
			2.28–2.74(n = 5)/2.83–3.38(n = 3)
Mid-M_3_	3.19	3.42	2.10–3.19
			2.10–2.65(n = 5)/2.58–3.19(n = 2)
**Corpus thickness vs. M_1_ size** b			
P_4_	1.56	1.80	-
M_1_ trigonid	1.39	1.69	1.14–1.37
			1.14–1.35(n = 5)/1.19–1.37(n = 4)
Mid-M_3_	1.87	2.67	1.75–1.91
			1.75–1.87(n = 5)/1.76–1.91(n = 4)
**Gracility index** c			
P_4_	54.10	54.60	-
M_1_ trigonid	46.45	48.85	38.73–58.97
			38.73–50.48(n = 5)/49.26–58.97(n = 3)
Mid-M_3_	58.59	78.20	60.04–84.07
			70.60–84.07(n = 5)/60.04–68.22(n = 2)

a depth/squareroot(M_1_ length*M_1_ breadth).

b thickness/squareroot(M_1_ length*M_1_ breadth).

c thickness/depth*100.

Corpus depths are means of buccal and lingual measurements. Corpus measurements and indices of *O. macedoniensis* are given with the total range on the first line and the intrasexual ranges (♀/♂) on the second line.

Another characteristic of MFI-171 is its low surface relief: the fossae for the masseter and the pterygoid muscles are shallow and the post C-P_3_ buccal depression is not well marked, unlike in TF 6223. On the lingual side of the jaw, the mylohyoid line is discernible. It starts from the inferior transverse torus and disappears under the M_3_. Below the mylohyoid line, the bone shows a shallow, narrow and short depression (submandibular fossa). In most other places, the bone is rather flattened. On the ventral and lingual margins of the corpus, no imprint for the anterior digastric muscle insertion is distinguishable (see Figures 2A, 2C and 2E) as on *K. piriyai* TF 6223 [4], [18]. The absence of impressions for the anterior digastric muscle bears a strong phylogenetic meaning because it is an exclusive feature of *Pongo* [19], [20] and *Khoratpithecus piriyai*. The corpus is extremely thin in the angular region (Table 1). Poorly preserved, the ramus originates at the level of the M_3_ and was probably hiding the distal part of its crown in lateral view. The buccinator groove is much narrower than in TF 6223.

**Figure 3:**
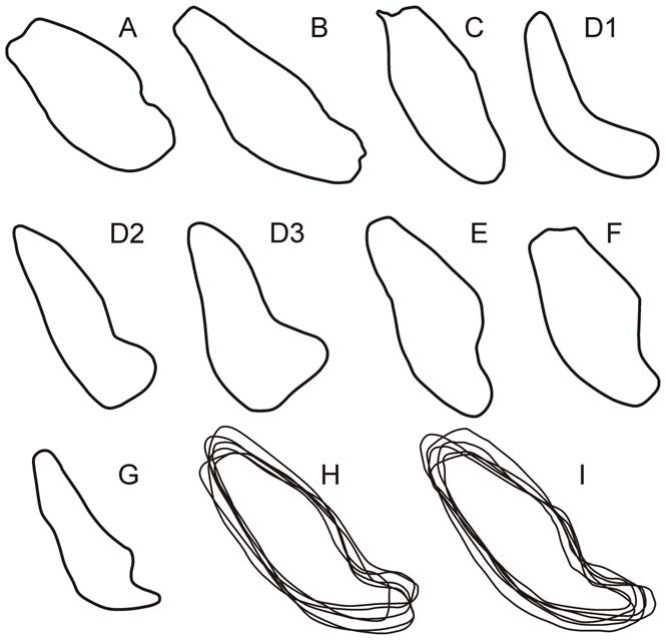
Comparison of the symphyseal sections of *Khoratpithecus ayeyarwadyensis* and selected fossil and extant hominoids. *Khoratpithecus ayeyarwadyensis* MFI-K171 (A), *Khoratpithecus piriyai* TF 6223 (B), *Indopithecus giganteus* (C), *Lufengpithecus lufengensis* adult female PA 895 (D1), young male PA 548 (D2) and adult male LC 102 (D3), *Sivapithecus sivalensis* GSP 9564 (E) and GSP 13875 (F), *Ankarapithecus meteai* female AS-95-500 (G), six symphyses of male (H) and female (I) *Pongo pygmaeus pygmaeus* aligned on their centroid. B from [18]; C, E, F, H, I from [20]; D from [21]; G from [37]. Symphyses not to scale. Note that *Lufengpithecus lufengensis* symphyseal sections are presumably deformed.

MFI-K171 is sufficiently well preserved in its anterior region to provide a good estimate of the symphyseal section outline. The specimen displays only a partial genial fossa because the break is not perfectly oriented along the midsagittal plane (Figures 2A, 2C and 3A). Due to the state of preservation of the symphysis, its posterior part does not extend medially to the midsagittal plane. A sagittal section of the corpus ∼1 mm lateral to the midsagittal plane was therefore used for reconstructing the posterior part of the symphyseal outline (Figure 3A).

The genial fossa is oriented distally and superior and inferior transverse tori are equally developed. MFI-K171 has a much shorter symphysis than TF 6223, especially at the level of the planum alveolare: the long axis in TF 6223 is 56% longer than that of MFI-K171. The inclination of the planum alveolare is similar between the two specimens.

**Figure 4:**
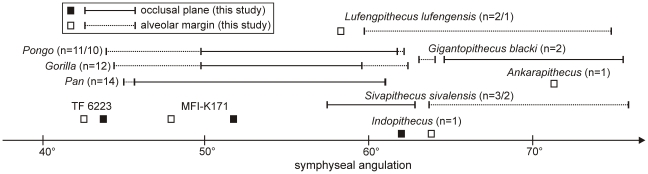
Comparison of the symphysis angulation of MFI-K171 with those of selected living and fossil hominoids. The angulation (measured between the long axis of the outline and alveolar margin plane and/or the occlusal plane) where obtained from CT-scans (*Pongo*, *Pan*, *Gorilla*, *Khoratpithecus piriyai* TF 6223, and MFI-K171) and pictures of casts (*Indopithecus*, *Gigantopithecus* AMNH 99528 and 39527, *Sivapithecus* GSP 15000, 9564 and 13875, *Lufengpithecus* PA 820 and 548, *Ankarapithecus*). The notation X/Y was used when the symphyseal angulations were measured between the long axis and the alveolar margin on X individuals and between the long axis and the occlusal plane on Y individuals.

The general outline of the symphyseal section of MFI-K171 is closer to that of TF 6223 than to any other Asian fossil hominoids, with subequal transverse tori, shallow genial fossa, and a supero-inferiorly convex anterior portion (Figure 3). Both MFI-K171 and TF 6223 also share a strong symphyseal inclination (respectively 48° and 42.5° to the alveolar margin of P_3_-M_3_) in contrast to *Sivapithecus*, *Indopithecus*, *Lufengpithecus*, and *Ankarapithecus* (Figure 4). The symphyseal section of MFI-K171 also differs from *Pongo* which displays more posteriorly directed and more elongated inferior transverse torus (Figure 3); from *Sivapithecus sivalensis* (GSP 9564 and GSP 13875) which have a thicker superior transverse torus relative to the inferior one and an impression for the anterior digastrics [19], [20]; from *Lufengpithecus lufengensis* (LC 102, PA 548, PA 895) which has a much larger inferior torus, especially in males (Figure 3), and an extensive anterior digastric muscle insertion scar extending to the ventral surface of the inferior torus (see fig. 3.20 in [21]); from *Lufengpithecus hudienensis* which possesses a posterior extent of the symphysis at the distal edge of the P_3_ (see PDYA122, also referred to as PDYV122, in [22], [23]); from *Ankarapithecus* which has an inferior torus which projects more posteriorly (Figure 3). The holotype mandible of *Griphopithecus alpani* MTA 2253 [24] also differs from MFI-K171 by a much stronger superior torus associated with a deeper genial fossa.

Only a few hominoids such as the African and Asian *Kenyapithecus* and perhaps the non-Asian *Equatorius* show comparable angulations of the symphysis [25]–[27]. *Kenyapithecus* differs from MFI-K171 by having a stronger superior torus relative to the inferior one.

In order to quantify the symphysis outline of MFI-K171 and compare it to those of other hominoids, we undertook an elliptic Fourier analysis on normalized Fourier coefficients, following the methodology of [28]. To perform a rigorous symphyseal outline analysis, only specimens for which CT-scans were available were utilized. Our comparative sample comprises TF 6223 and adult specimens of *Pongo pygmaeus* (n = 15, 8 males and 7 females), *Gorilla gorilla* (n = 32, 19 males and 13 females), *Pan troglodytes* (n = 44, sex-balanced), *Pan paniscus* (n = 11, 4 males and 7 females) and *Homo sapiens* (n = 27, 15 males and 12 females).

**Figure 5:**
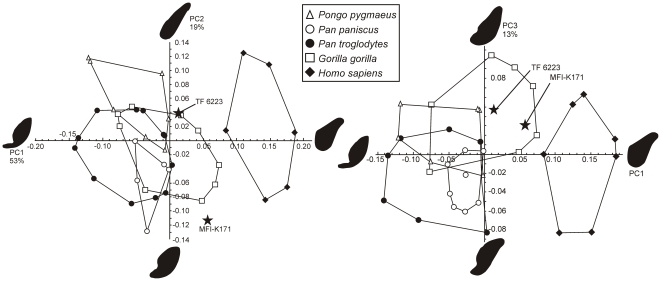
Principal component analysis plots of the elliptic Fourier coefficients of the symphyseal outlines of *Khoratpithecus* MFI-K171 and TF 6223 and extant hominoids. Only the outlying individuals are represented for the extant hominoids. For each component, extreme outlines and expressed variance are given.

A principal component analysis (PCA) was then performed on the Fourier coefficients. The PCA yielded seven significant axes expressing 95% of the total variance. The first three components represent 85% of the total variance (respectively 53%, 19%, and 13% of the total variance). The four other significant components represent in total 10% of the total variance (3.6%, 3.1%, 1.8% and 1.3%). Figure 5 represents the PCA scores corresponding to the symphyseal outlines plotted in the PC1-PC2 and PC1-PC3 spaces. PC1 represents a deviation from circularity: high values on PC1 yield rather circular outlines characterized by thick inferior transverse torus, a very weak genial fossa, a long, flat, and weakly inclined planum alveolare, and a weakly concave anterior border. In contrast, low PC1 values yield more outlines deviating from circularity and characterized by very deep genial fossa, thin and distally elongated inferior transverse torus, and convex antero-inferior and postero-superior borders. PC2 describes the relative elongation of the outline, the convexity of the antero-inferior and postero-superior borders and, to a lesser extent, the depth of the genial fossa. High PC2 scores involve outlines with great length/thickness ratio and flat antero-inferior and postero-superior borders, while low scores are represented by thicker symphyses with a distinct genial fossa, and convex antero-inferior and postero-superior borders. PC3 differentiates outlines with subequal tori, long planum alveolare and convex antero-inferior border (high scores) versus outlines with thicker superior torus, convex postero-superior border with short planum alveolare, and slightly concave antero-inferior border (low scores). The PCA distinguishes *Homo sapiens* symphyses (high scores on PC1) from those of other hominoids. Modern hominoids symphyseal patterns can be differentiated in the PC1-PC2 space though significant overlap between the domains of variation exists. In this space, TF6223 (*K. piriyai*) lies just outside the domain of *Pongo*, having a thicker symphysis with a thicker and less posteriorly projected inferior torus (higher PC1 score). TF 6223 also lies just outside to the domain of *Gorilla*, which is characterized by scores close to 0 on PC1 and PC2. MFI-K171 falls outside the clusters of all living taxa. It is also noticeably distant from TF 6223 in the PC1-PC2 space, mostly because of a great difference in the PC2 score. This difference is explained by the more pronounced genial fossa, the more convex anterior border and much less proportionally elongated symphyseal outline of MFI-K171. In the PC1-PC3 space, the living taxa have distinct outline patterns with partial overlap. MFI-K171 and TF 6223 are close to each other and lie within the domain of *Gorilla*, which is characterized by mostly positive scores on PC3 and score close to 0 on PC1. The presence of subequal tori in both TF 6223 and MFI-K171 explain their close scores on PC3. A higher PC3 score for TF 6223 can be explained by its longer planum alveolare and weaker genial fossa relative to MFI-K171.

Given the substantial distance between the two *Khoratpithecus* mandible in the PC1-PC2 space, we computed the intraspecific distance matrix in the principal component space for the living hominoids and compared it to the MFI-K171-TF 6223 distance. For this analysis, we chose Euclidian distances computed between pairs of individuals in each sample with the seven significant components. The result of this analysis is that the distance between MFI-K171 and TF 6223 (d = 0.17) falls in the upper limit of the intraspecific variability of modern hominoids: the frequencies of distances exceeding 0.17 are 3.9% in *Pongo pygmaeus* (4/105), 2.2% in *Gorilla gorilla* (11/496), 0.6% in *Pan troglodytes* (6/946) and 0% in *Pan paniscus* (0/55). This analysis shows that conspecificity between MFI-K171 and TF 6223 is unlikely.

Incisor alveoli are deep and possess ovoid sections with a labio-lingual long axis and a mesio-distal compression. Both incisor alveoli bear small absolute dimensions (Table 3). The incisors alveoli of *Khoratpithecus ayeyarwadyensis* can be distinguished from those of *K. piriyai* by their greater proclination, their smaller size and their more pronounced narrowness (according to [18], TF 6223 I_1_ alveoli average 8.4 by 5.9 mm against 5.8 by 2.2 mm for MFI-K171 while TF 6223 alveoli of the left and right I_2_ measure respectively 10 by 5.8 mm and 8.4 by 4.3 mm against 7.6 by 3.2 mm for MFI-K171). The last two differences reflect an important disparity in width of the alveolar incisor area (measured between the mesial edge of the I_1_ alveolus and the distal edge of the I_2_ alveolus): 8.86 mm for MFI-K171 against 13.83 mm for TF 6223 [18] although M_1_s in the two specimens are of similar size.

**Table 3:** Dental measurements of *Khoratpithecus ayeyarwadyensis* (in millimeters).

Teeth and alveoli	Mesio-distal length	Bucco(labio)-lingual length (trigonid/talonid)	Height
I_1_ (alveoli)	2.19	5.78	-
I_2_ (alveoli)	3.17	7.6	-
C (alveoli)	6.45	8.05	-
P_3_	13.5	9.68	8.89 (Protoconid)
P_4_	8.19	9.92	7.30 (Protoconid)
M_1_	12.29	11.05/10.33	5.91 (Metaconid)
M_2_	13.92	12.75/11.46	6.15 (Metaconid)
M_3_ (dentine section)	14.2	11.93/9.25	-

Incisor comparisons between MFI-K171 and *K. chiangmuanensis* are limited because the hypodigm of the latter species contains only a single I_1_, TF 6178 (see Fig. 3k in [3]). TF 6178 preserves a small portion of root very close to the cervix, which dimensions are >3 mm (mesio-distal) by 6.35 mm (labio-lingual).

The canine alveolus revealed by X-ray microtomography has a substantial volume in comparison to the roots/alveolus of the other teeth (Figure 2I–J). The length of the preserved alveolus measured in a sagittal plane is 23.2 mm from the apex to the barycentre of the preserved alveolar outline (Figure 2I–J). This barycentre is a few millimetres below the alveolar margin of the incisors and more than 6 mm below the alveolar margin of P_3_-M_3_ in a sagittal plane. Thus, taking into account the damage to the alveolus and the position of the crown cervix a few millimeters above the alveolar margin, the corresponding canine root was longer, probably approaching 30 mm. This size of canine root, along with its considerable transverse section, indicates that MFI-K171 was most likely a male, females having distinctly shorter canine roots in fossil and extant hominoids: the range of male canine root length is 27.6–36.6 mm for the Paşalar hominoids *Kenyapithecus kizili* and *Griphopithecus alpani* [29]. The roots of the two male canines of *Proconsul nyanzae* KNM-RU 2034 and KNM-RU 1676 measure, respectively, 29.2 and 32.5 mm (Kelley, pers. comm., 2010). In the male *Khoratpithecus piriyai*, TF 6223, the root of the right canine measures 31.9 mm. The smaller-sized *Proconsul nyanzae* excepted, the above-cited hominoids can be compared with MFI-K171 since they are in the same range of molar size. The anterior root of the P_3_ is strong and curved buccally, common features in male hominoids and associated with the strong canine root.

The canine alveolus has a triangular outline with rounded corners, comparable to that of *K. piriyai*. However, the alveolus is smaller sized and shallower (estimated alveolus length of MFI-K171 shorter than root length of TF 6223 canines; maximum dimensions of the alveolus is 6.45 by 8.05 mm in MFI-K171 while the maximum alveolus dimensions in TF 6223 is greater than those of the left broken canine: 10.11 by 13.78 mm), narrower and more proclined than those of TF 6223 (see fig. 5 in [18]). Judging from the position of the canine alveolus, there was only a very short or even no diastema between this tooth and the I_2_. A small contact facet on the mesial face of the P_3_ shows that no diastema was present between C and P_3_. This condition contrasts with the presence on TF 6223 of a pre- and a post-canine diastema which both measure about 2.5 mm.

The P_3_ is a sharp tooth (height/mesio-distal length = 0.66) dominated by a large and moderately elevated protoconid whose buccal face is distinctly slanted. The lingual face of the protoconid is rectilinear and shows an angle of about 30° to the dental row axis. It displays a low and thin cingulum between a small and low parastyle and the disto-lingual border of the tooth. The preprotocristid is mesially oriented and ends into the parastyle. The metacristid lacks a distinct metaconid and becomes bifurcated distally. The postprotocristid, only slightly worn, is curved lingually to the disto-lingual border of the tooth. The talonid basin corresponds to a narrow and shallow furrow between the postprotocristid and the metacristid. A narrow honing facet is observable on the mesiobuccal side of the tooth. It extends over the entire height of the crown. The P_3_ of MFI-K171 is different from those of *Sivapithecus sivalensis*, *Lufengpithecus keiyuanensis* and *L. hudienensis* [23] which have a less buccally slanted protoconid and less rotated lingual walls of the protoconid relative to the antero-posterior axis. *Lufengpithecus lufengensis* (PA 548, 580, PA 848, PA 895) has more similar proportions and slanted protoconid but differs by a much wider and deeper talonid basin and an incipient metaconid. Species of *Lufengpithecus* also have higher crowns due to greater heights of the protoconid. MFI-K171 shares with all the P_3_s attributed to *Khoratpithecus* the buccal bulge of the protoconid and, with the P_3_s of TF 6223, the narrow and deep talonid basin. Nonetheless, the P_3_ of MFI-K171 differs from those of *K. chiangmuanensis*, which shows a much larger distal basin and a more slanted protoconid, and from those of TF 6223 by its complete lingual cingulum and its shorter mesio-distal length. The difference in size of the distal basin between the P_3_s of MFI-K171 and the subcomplete P_3_ of *K. chiangmuanensis* TF 6171-7 can be quantified by the calculation of the ratio between the distal basin surface area (including the contribution of the distal face of the protoconid) and the maximum crown surface area. This ratio is 0.121 for MFI-K171 against 0.199 for TF 6171-7 while a ratio of 0.183±0.036 (N = 21) was obtained in a sample of *Pongo pygmaeus* (this study). Thus, there is reasonably a difference in distal basin development between MFI-K171 and TF 6171-7, the quantified difference (0.078) being greater than two standard deviations of the *Pongo* sample.

The P_4_ has a large and buccally-bulged protoconid and a smaller metaconid separated by a shallow mesio-distal groove. The metaconid is slightly mesial to the protoconid. The talonid is large, closed, and lower than the trigonid. The postprotocristid joins a distolingual crest which closes the deep talonid basin by joining the metaconid. There is no cingulid. The talonid of the P_4_ of MFI-K171 is higher in relation to the trigonid than those of *Lufengpithecus keiyuanensis* (pl. 1 and fig. 4a in [30] and pl. 1, figs. 2 and 4 in [31]), which also possesses a very shallow talonid basin. P_4_s of *Lufengpithecus lufengensis* (PA 548, PA 848, PA 580, PA 895) can be differentiated by more widely separated apices of protoconid and metaconid, larger and deeper talonid basin, and larger anterior basin. Specimens of *Lufengpithecus hudienensis* (PDYA112, PDYA108, PDYA122, PDYA208; see [22], [23]) are dissimilar to MFI-K171 in having less buccally-bulged crowns, a larger talonid basins, and possibly a lower talonid relative to the trigonid (feature noted on PDYA122). P_4_s of other species of *Khoratpithecus* share with MFI-K171 a buccally-bulging protoconid, a protoconid and a metaconid close together, and a deep and completely closed talonid basin.

The P_4_ of MFI-K171 can be distinguished from those of *K. chiangmuanensis* by a lesser relative size of the talonid basin: the ratio between the surface area of the talonid basin (including the contribution of the distal faces of the protoconid and the metaconid) and the maximum crown area is for 0.333 for MFI-K171 against 0.459 and 0.456 for *K. chiangmuanensis* TF 6171-3 and TF 6179. In a sample of *Pongo pygmaeus* (this study), a ratio of 0.382±0.043 was obtained (N = 21). The difference between MFI-K171 and the mean of the P_4_s of *K. chiangmuanensis* molars is 0.125, which represents about three standard deviations of the *Pongo* sample. Thus, it is reasonable to consider that the P_4_s of MFI-K171 and *K.chiangmuanensis* differ in terms of development of distal basin. In addition, the mesiodistal∶buccolingual ratio of MFI-K171 is 0.83 against 0.94 for TF 6171-3 and 0.91 for TF 6179. This difference is important but not greater than two standard deviations in a *Pongo* sample (ratio = 1.01±0.07, N = 22; this study) and seems to be caused by a more slanted protoconid in *K. chiangmuanensis*. A mesial position of the metaconid relative to the protoconid, feature present on TF 6223, and a much less marked valley between the protoconid and the metaconid are additional features that distinguish the P_4_ of MFI-K171 from those of *K. chiangmuanensis*. P_4_s of TF 6223 have stronger and higher talonids, distinct hypoconid and entoconid, and shallower talonid basins.

Only remnants of enamel wrinkling are visible on the M_1_ because of its stage of wear (small dentine pits apparent on the protoconid and hypoconid). The buccal face of the tooth is markedly slanted unlike in TF 6223, *Sivapithecus sivalensis* (GSP 9564, GSP 15000, GSP 16082), *Lufengpithecus lufengensis* (PA 548, PA 848, PA 580, PA 895) and *L. keyuanensis* (pl. 1 in [31]). The trigonid is wider than the talonid (Table 3). In *Sivapithecus* and *Lufengpithecus* trigonid and talonid have generally subequal breadths. The M_1_s of *Khoratpithecus piriyai*, which are proportionally slightly more elongated, show an opposite pattern with broader talonids. The hypoconulid is strong. There is no buccal cingulid. The central fovea seems deeper than in *Sivapithecus* and as deep as in *Lufengpithecus* at similar wear stages. Although the tooth is worn, it was possible to estimate the ratio between the surface area of the central fovea and the maximum crown surface at 0.35.

The M_2_ of MFI-K171, nearly unworn, shows strong cusp relief, markedly wrinkled enamel, and is substantially wider in the trigonid region than in the talonid as in the M_1_. The wrinkling is better expressed around the large central fovea (in particular on the medial face of the entoconid which shows three furrows) and between the protoconid and the metaconid. As on the M_1_, there is no buccal cingulid. This combination of features is different from that of *Sivapithecus* on which M_2_s have flatter buccal sides, nearly constant breadths as well as weaker cusp relief; from *Lufengpithecus lufengensis* and *L. keyuanensis* which have less slanted cusps and very weak ectoflexid and from those of *L. hudienensis* which also possess weak ectoflexids and frequently display broader talonids on both M_1_ and M_2_ [22]. This M_2_ is distinguishable from those of *K. chiangmuanensis* by a smaller central fovea: the ratio between the surface area of the central fovea and the maximum crown surface area is 0.367 in MFI-K171 (which is close to the value estimated for the M_1_) against 0.426 for mean of the two unworn molars of *K. chiangmuanensis*. In a sample of *Pongo pygmaeus* (this study), a ratio of 0.427±0.027 was obtained (N = 25). The difference between MFI-K171 and the molars of *K. chiangmuanensis* molars is 0.595, which represents slightly more than two standard deviations of the *Pongo* sample. Thus, it is reasonable to consider that the M_2_ of MFI-K171 is different in terms of development of the central fovea from the specimens of *K. chiangmuanensis*. The M_2_ of MFI-K171 has also a greater width of the trigonid relative to the talonid (the trigonid breadth∶talonid breath ratio is 1.11 for MFI-K171 against 1.07 in average for the three M_2_s of *K. chiangmuanensis*), and a stronger ectoflexid. The M_2_s of *K. piriyai* differ from those of MFI-K171 by a quadratic outline in occlusal view due to subequal breadth of the trigonid and the taloind, more vertical faces of the buccal cusps and an incipient buccal cingulid.

The M_3_ has a triangular section at the cervix. The surface area of this tooth at the cervix is similar to the M_1_ crown surface area (at the cervix) and about 22% smaller than that of the M_2_ (at the cervix). Nevertheless, it is reasonable to consider that the crown of the M_3_ was close in size to that of the M_2_.

The roots of MFI-K171 (Figure 2I–J) are extremely similar to those of *K. piriyai* (see fig. 5 in [18]) in terms of relative size, length, orientation and curvature. The incisor roots of the Burmese mandible are short, narrow mesio-distally, broad bucco-lingually, and show procumbent inclination close to those of TF 6223. The canine alveolus is smaller and shorter than the canine root of TF 6223 but shares a similar inclination. The anterior root of the P_3_ is very strong, curved distally, and buccally positioned. The two roots of the P_3_ are only slightly divergent. The roots of the P_4_ are gracile, sub-parallel, and nearly as long as those of the P_3_. Molar roots are shorter than those of the premolars, slightly divergent and rather straight. Even if root morphology often displays greater variation than the corresponding crown, the resemblance with the molar radicular pattern of *K. piriyai* is very striking.

#### Specific attribution of MFI-K171

The combination of features displayed by the mandible MFI-K171 (absence of an imprint for the anterior digastric muscle, the symphyseal outline and inclination, and the association of certain dental traits such as buccal slanted protoconids on P_3_ and P_4_, narrow and deep talonid basin on P_3_, deep and closed talonid basin on P_4_, protoconid and metaconid close together on P_4_, deep central fovea on molars) excludes any close affinity with the Asian Neogene hominoid genera *Sivapithecus*, *Lufengpithecus*, *Indopithecus*, *Gigantopithecus* and *Ankarapithecus*. Conversely, they justify the allocation of the specimen to the genus *Khoratpithecus*, formerly only known in the Miocene of Thailand [3], [4].

Although the teeth of MFI-K171 and the *K. piriyai* holotype, TF 6223, are nearly of the same size (except the M_3_), the two specimens have very different mandibular anatomies: the corpus is robust with a great intercanine breadth, a long symphysis, and a strong lateral eminence at M_3_ for TF 6223 while it is slender with a much shorter intercanine breadth, a shorter symphysis and a weak lateral eminence at M_3_ for MFI-K171. Besides metrical aspects, the symphyses of MFI-K171 and TF 6223 have also distinctive outline patterns. The distance in the Fourier morphological space between the two outlines is substantial and can only be observed within extant hominoid species at low frequencies, rendering the conspecificity of the two specimens unlikely. In addition, the several dental dissimilarities that exist between MFI-K171 and TF 6223 in the premolars (especially the P_4_), the flaring of the buccal walls of molars, and the relative size of the M_3_ all suggest that MFI-K171 cannot be assigned to *Khoratpithecus piriyai*.

Numerous dental differences in the molars (smaller central fovea) and premolars (smaller distal basin and less slanted protoconid on the P_3_; P_4_ with a smaller talonid basin, a lower mesiodistal∶buccolingual ratio) support a specific distinction between MFI-K171 and the material assigned to *K. chiangmuanensis* and justify the erection of a new species name for MFI-K171.

### *Khoratpithecus* sp

#### Comparative description

**Figure 6:**
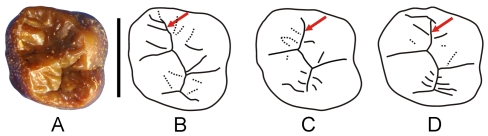
MFI 89, upper molar of *Khoratpithecus*. A: right M^2^ MFI89 in occlusal view. B: interpretive drawing of MFI89. C–D: interpretive drawings of *Khoratpithecus chiangmuanensis* left M^2^ TF 6176 (mirror image) and right M^2^ TF 6169. The upper arrows point the valley between the paracone and the protocone which is deviated in MFI89. Scale bar: 1 cm. C–D are not to scale.

**Figure 7:**
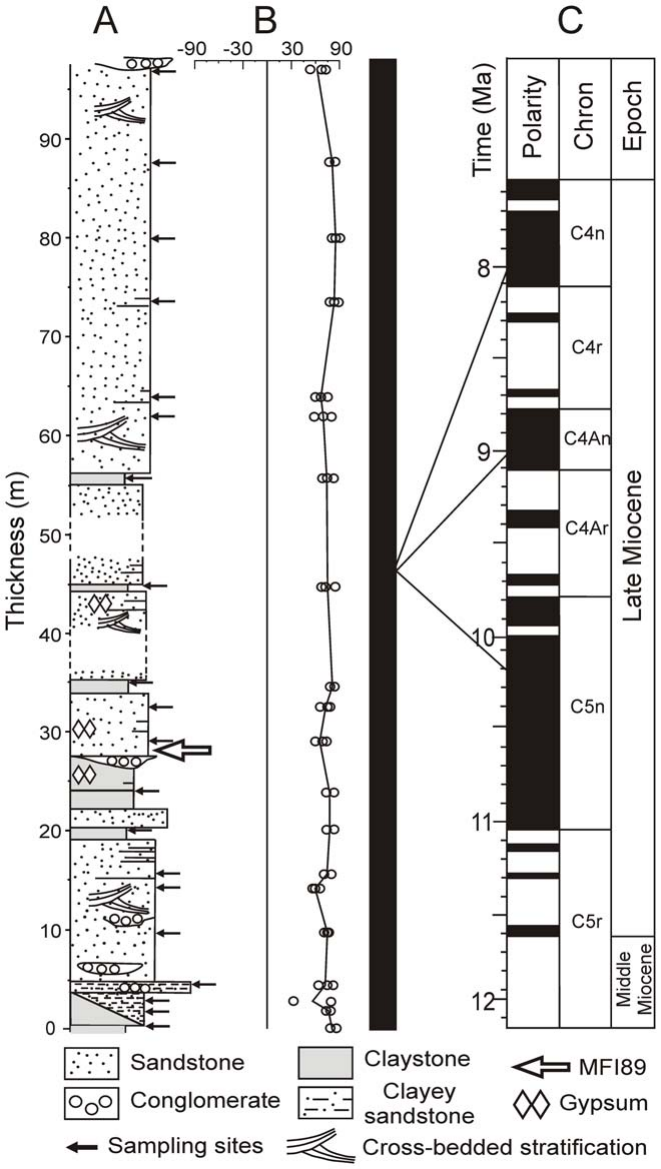
Magnetic polarity stratigraphy of the hominoid section. A: lithology and stratigraphic position of the sampled levels. B: latitude of the virtual geomagnetic pole vs. stratigraphic position. C: polarity column (black bar: normal polarity zone) and potential correlations with the GPTS of [35].

MFI 89 (Figure 6A–B) is a complete M^2^ crown of a large sized hominoid. It was unearthed by the Myanmar-French paleontological expedition in december 2007 from a channel deposit rich in iron-hydroxides in a nodular layer associated with several other mammal teeth (located 20°06′35.43″N 95°06′42.47″E). Geologically, its corresponding section is mainly composed of a succession of light-coloured sands and gray clays, with several ferruginous conglomerate nodules (Figure 7).

MFI89 is close in size to M^2^s of presumed male *Khoratpithecus chiangmuanensis* (length: 13.61 mm; breadth: 13.83 mm; height (protocone): 9.21 mm.) but slightly smaller than the corresponding lower tooth in the holotype of *K. ayeyarwadyensis*. The M^2^ is nearly unworn, only small facets being perceivable on the summit of the main cusps (mostly on the metacone). This tooth displays strong and abundant enamel wrinkling, most of which is concentrated on its anterior part. The protocone is very large while the paracone is reduced and low. The groove between these two cusps is oblique (Figure 6). The hypocone is developed. The distal fovea is large. The occlusal surface is close to a square with a mesiodistal to buccolingual width ratio of 0.98. The crown is high.

The cusp organisation of MFI 89 strongly resembles that of the M^2^s of *K. chiangmuanensis* except that the Thai specimens differ by displaying a mesiodistal groove between the protocone and the paracone (Figure 6). From a biometrical point of view, MFI 89 is extremely close to the mean value of *K. chiangmuanensis* in terms of length/width proportions.

#### Taxonomic attribution

The molar MFI 89, because of its strong resemblance with *K. chiangmuanensis* is referred to the genus *Khoratpithecus*. It shares with the latter genus the slanted buccal and lingual cusps, and a sharp, strong and elevated metacrista. It only differs from the upper molars of *K. chiangmuanensis* by the orientation of the groove between the protocone and the paracone, and higher crown elevation. Due to the isolated nature of the specimen, it seems premature to assign this tooth a particular species, although its belonging to *K. ayeyarwadyensis* is suspected.

### Chronology of the hominoid-bearing deposits

#### Magnetostratigraphy

**Figure 8:**
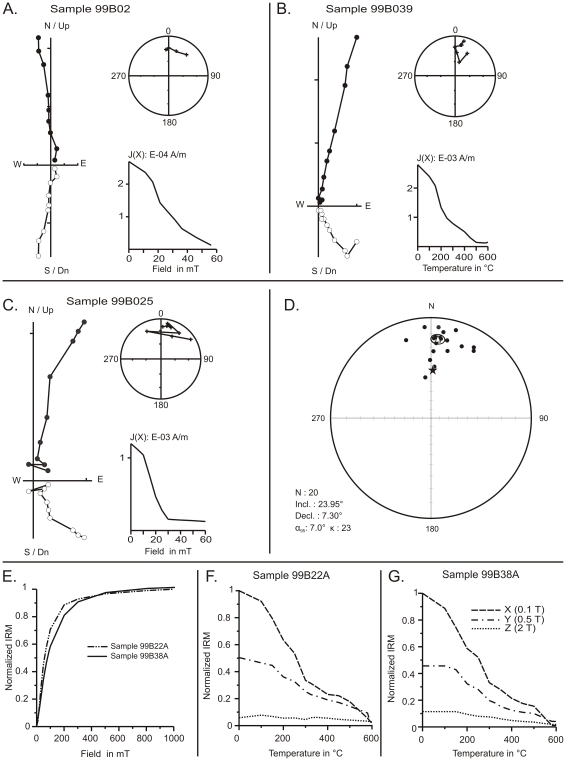
Paleomagnetic analyses of the samples from the hominoid section. A–C: Orthogonal vector diagrams (closed/open symbols correspond to the horizontal/vertical component), stereoplots (crosses: upper hemisphere, circles: lower hemisphere), intensity and step plots for representative samples after thermal and alternating field demagnetization. D: Equal-area stereographic projections of site mean ChRM directions. Open triangle: site mean direction (Declination = 7.3°; Inclination = 23.9°; α_95_ = 7; k = 23; n = 20), Ellipse: 95% confidence ellipse for mean direction. Solid star: direction derived from the 10-Ma apparent polar wander path. E: Isothermal remanent magnetization (IRM) acquisition curves (normalized values) for two representative samples. F–G: Stepwise thermal demagnetization of the differential IRM components (X, Y, and Z). 99B022A: medium grained sandstone. 99B039A: grey claystone.

A magnetic polarity stratigraphy of the section of MFI89 was established on 20 distinct levels along a 97 m thick section (Figure 7). Paleomagnetic analyses were carried out at the laboratory of paleomagnetism of the Universidad Nacional Autonoma de Mexico. Rock magnetic analyses indicate a uniform magnetic mineralogy along the section and reveal that the ferromagnetic carriers of remanent magnetization are mainly titanomagnetite, magnetite, to a lesser extend, hematite and some iron-sulfides (Figure 8E–G). The intensity of the remanent magnetization is one order of magnitude higher in grey claystone than in sandstones samples. Alternating field and thermal demagnetization isolated successfully the different components of magnetization and allowed an unequivocal determination of the Characteristic Remanent Magnetization (ChRM) directions (Figure 8). The ChRM directions were calculated by principal component analysis [32]. Mean directions and associated parameters (k, α_95_) were calculated for each level using Fisher [33] statistics (Table 4). ChRM mean directions after stratigraphic correction are: Decl. = 7.30° and Incl. = 23.95° (k = 23, α_95_ = 7°). ChRM declination is not significantly different, at a 95% level of certainty, to the expected paleofield declination calculated from the 10-Ma apparent polar wander path [34]. Virtual geomagnetic pole paleolatitudes, calculated from ChRM directions, yielded a normal magnetic polarity sequence for our whole section. Age constraints for the correlation of the magnetic polarity stratigraphy to the GPTS [35] are provided by biochronological data.

**Table 4:** Site-mean ChRM directions and associated virtual geomagnetic pole paleolatitude.

Site	Height(m)	Incl.	Decl.	α_95_	k	λ_VGP_
1	0	56.32	351.64	-	-	79.14
2	2	22.41	11.65	-	-	75.78
3	3	19.31	31.26	-	-	55.25
4	4.5	41.62	358.83	17.7	49	86.43
5	9.5	10.06	4.68	14.0	79	74.28
6	14.2	21.81	32.32	17.3	52	57.73
7	15.5	14.47	8.07	13.5	84	75.03
8	20	20.49	3.25	13.2	88	79.97
9	24	20.49	6.63	14.2	76	78.53
10	28.5	9.89	355.82	23.3	29	74.33
11	32.5	2.17	5.07	13.4	85	70.34
12	35	31.7	13.65	-	-	76.50
13	45	47.39	3.51	9.0	190	80.99
14	60	3.74	15.69	9.8	160	66.18
15	62	33.67	2.28	9.7	164	87.26
16	64	23.24	23.05	19.1	43	66.49
17	73.5	23.37	4.24	18.9	44	81.09
18	82	22.56	2.98	15.4	65	81.15
19	87.5	21.8	1.75	-	-	81.17
20	97	19.43	342.07	41.7	10	70.00

Height: stratigraphic height above the first level sampled; Incl.: mean tilt corrected inclination in degree to the horizontal; Decl.: mean tilt corrected declination in °E; α_95_: mean direction 95% confidence ellipse; k: Fisher precision parameter; λ_VGP_: paleolatitude of the virtual geomagnetic pole.

#### Biochronology

The associated mammal fauna collected during our fieldwork is homogeneous along the Magway sections and among the investigated area. It includes a proboscidean belonging to the genus *Tetralophodon*, the equid *Hipparion* (*s.l.*), a species of Chalicotheriinae and an indeterminate species of rhinocerotid. Artiodactyls are represented by the anthracothere *Merycopotamus medioximus*, the suids *Tetraconodon minor* (Tetraconodontinae) and *Propotamochoerus* cf. *hysudricus* and cf. *Hippopotamodon sivalense* (Suinae), the palaeochoerid *Schizochoerus* sp., a medium-sized tragulid, three bovids including an antelope, and a giraffid. A fresh water neoselacian, numerous trionychid plate fragments, crocodile dental and skeletal remains and siluriform fishes were also discovered in the MFI89 section. The complete faunal list is given in Table 5.

**Table 5:** Faunal list of the Magway section.

MAMMALIA
**Primates**
Hominidae
*Khoratpithecus ayeyarwadyensis*
**Proboscidea**
*Stegolophodon/Tetralophodon* sp.
**Artiodactyla**
Suidae
*Tetraconodon minor*
cf. *Hippopotamodon sivalense*
*Propotamocheorus* cf. *hysudricus*
Palaeochoeridae
*Schizochoerus* sp.
Tragulidae
Gen. et sp. indet. (medium-sized)
Bovidae
Antilopini indet.
Gen. et sp. indet.
Gen. et sp. indet. 2
Anthracotheriidae
*Merycopotamus medioximus*
**Perissodactyla**
Equidae
*Hipparion (s.l.)* sp.
Rhinocerotidae
Gen. et sp. indet.
Chalicotheriidae
Chalicotheriinae
Gen. et sp. indet.
**Carnivora**
Fam. Gen. et sp. indet.
REPTILIA
**Testudines**
Trionychidae
*Trionyx* sp.
CHONDRYCHTHYES
**Carcharhiniformes**
Carcharhinidae
*Glyphis pagoda*
ICHTHYES
**Siluriformes**
Fam. Gen. et sp. indet.

This associated fauna, among which hipparionin fossils are most common, is younger than 10.7 Ma, the observed first appearance datum (FAD) of these equids in the Siwaliks of Pakistan [36]. This FAD is consistent with that observed in the Turkish Sinap Formation (10.7 Ma; see [37]). The anthracothere *Merycopotamus medioximus* appears to have evolved in the Indian subcontinent from the species *Merycopotamus nanus* during the early late Miocene [38]. *M. medioximus* ranges from 10.4 to 8.6 Ma in the Siwaliks of Pakistan. Although a short temporal gap exists between the ranges of *M. nanus* and *M. medioximus* [39], the observed FAD of the latter species in Pakistan confirms the indication of hipparionins. The tetraconodont *Tetraconodon minor* is a close relative of *Tetraconodon magnus* present between 10.0 and 9.3 Ma in Pakistan [36]. Moreover, the MFI89 section yielded two primitive Suinae (*Propotamochoerus* cf. *hysudricus* and cf. *Hippopotamodon sivalense*) which match best with the two dominating suids of the late Miocene fauna of the Indian subcontinent, *Propotamochoerus hysudricus* and *Hippopotamodon sivalense*. These species are associated together from 10.2 to 7.2 Ma in the Potwar Plateau of Pakistan [36].

Because the Burmese fauna includes several shared or closely-related species with the Siwaliks of Pakistan, the first and last appearance data (LAD) of the Pakistani taxa are used to provide the biochronological bracket. This choice is also motivated by fossil richness and chronological precision of the sites of this region [36]. As mentioned above, the FADs of the Siwalik species bring a convincing lower limit for the age bracket of the MFI89 section. Uncertainty is greater for the upper bracket, the LADs of the Siwalik large mammals being not well-constrained in Southeast Asia [18]. Considering the biochronological constraints, an optimized bracket of 10.4–8.6 Ma is assigned to the MFI89 section. Within the early late Miocene, three normal polarity chrons (C5n, C4An and C4n.2n) can fit with our magnetic polarity section. Correlations with shorter normal chrons, implying very high sedimentation rates, can be excluded [40], [41]. The correlation with subchron C4n.2n (8.1–7.7 Ma) is less probable taking into account the biochronological data, which rather support allocating the MFI89 section either within chron C4An (9.1 to 8.8 Ma) or within the latest part (∼10.4–9.8 Ma) of chron C5n.

### Preliminary paleoenvironmental analysis

The stable isotopes from enamel and dentine of 6 *Hipparion* (*s.l.*) samples of distinct layers from the MFI89 section were selected and analyzed. A diamond drill bit was used to obtain 5 to 10 mg powder from enamel, cement, dentine or bone tissues. All the samples were chemically pre-treated with 2% NaOCl solution, followed by a 1 M Ca-acetate acetic acid buffer solution [42], prior to analysis of the carbon and oxygen isotopic composition of the carbonate in the phosphate. C- and O- isotope composition was determined using a gasbench II device connected online with a Finnigan MAT 252. Isotope ratios of samples are calibrated using NBS18 (δ^13^C = −5.00‰, δ^18^O = −22.96‰, relative to VPDB) and NBS19 (δ^13^C = 1.95‰, δ^18^O = −2.20‰, relative to VPDB). External reproducibility is better than ±0.1‰ for δ^13^C and ±0.1‰ for δ^18^O measurements. δ^18^O_SMOW_ values were also calculated to allow comparison with other published data using the formula δ^18^O_SMOW_ = 30∶91+1.03091 δ^18^O_PDB_ [43].

Tooth enamel of large herbivores such as *Hipparion* (*s.l.*) is considered relatively stable towards diagenetic alteration for millions of years due to their very high degree of mineralization [44], [45]. In the present case, the carbonate content of *Hipparion* (*s.l.*) tooth enamel is within the range of values measured for modern tooth enamel (2.3 to 5.1%) [46], [47]. In addition, the use of continuous flow system of CO_2_ purification avoids possible interferences of iron and manganese oxydes in the C and O isotopic determination [48]. In contrast to enamel, other skeletal tissues such as bone, dentine and cement exhibit much less stability towards diagenetic alteration. Their carbon and oxygen isotopic signatures usually do not reflect those of the living animal [49], [50].

The δ^13^C values obtained from tooth tissues and dentary bone of *Hipparion* (*s.l.*) range from −15.3 to −9.9‰ (Table 6) and are all clearly lower than the threshold value of −8‰, above which contribution of C4 biomass becomes significant [51]. Enamel δ^13^C range from −12.1 to −9.9‰ and indicate the consumption of a large proportion of C3-plants [52]. The carbon isotopic values of cement, dentine and bone range from −15.3 to −13.1‰ (Table 6) and point to a rather dense canopy forest. The δ^18^O values of *Hipparion* (*s.l.*) enamel are lower than those measured on this taxon in the Siwaliks [53], [54] and indicate humid conditions, as do the low δ^18^O values measured on dentine, cement and bone (Table 6), since the ^18^O content of precipitation decreases with precipitation amount in inter-tropical areas [55].

**Table 6:** δ^13^C and δ^18^O values obtained from six *Hipparion (s.l.)* specimens from the hominoid section.

Specimen	Tissue	CaCO_3_(**%**)	δ^13^C_PDB_(‰)	δ^18^O_PDB_(‰)	δ^18^O_VSMOW_(‰)
IRWD 1	Enamel	4.0	−9.9	−3.9	26.9
IRWD 2	Enamel	4.0	−11.4	−4.3	26.4
IRWD 3	Enamel	4.9	−12.1	−4.9	25.8
IRWD 4	Enamel	4.4	−11.7	−6.8	23.8
IRWD 5	Enamel	5.2	−11.0	−8.8	21.8
IRWD 6	Enamel	3.6	−11.1	−4.5	26.2
**Mean**		4.3	−11.2	−5.5	25.2
**Standard deviation**		0.6	0.7	1.7	1.8
IRWD 1	Dentine	7.3	−13.1	−7.4	23.2
IRWD 2	Cement	6.8	−14.4	−6.7	24.0
IRWD 3	Cement	5.3	−15.3	−8.5	22.1
IRWD 4	Bone (dentary)	9.0	−14.3	−6.6	24.0
IRWD 5	Dentine	6.7	−13.7	−8.0	22.6
IRWD 6	Dentine	6.1	−13.6	−7.6	23.0
**Mean**		6.9	−14.1	−7.5	23.2
**Standard deviation**		1.1	0.7	0.7	0.7

### Conclusions

The genus *Khoratpithecus* is known from two others localities in Thailand; with *Khoratpithecus chiangmuanensis* in Chiang Muan basin in Northern Thailand [3] and *Khoratpithecus piriyai* in Nakorn Ratchasima Province, in Northeastern Thailand [4], [18]. Chiang Muan basin was dated between 13.5 and 10 Ma [56], [57], the hominoid layers being dated between 12.4 and 12.2 Ma, according to recent correlations [58] and *K. piriyai* was discovered in Late Upper Miocene sediments [4], [18].

New specimens discovered in the Irrawaddy Formation, in Central Myanmar, document a new species of the large bodied hominoid genus *Khoratpithecus*, *K. ayeyarwadyensis*. Magnetostratigraphic and biochronological data indicate an age of either ca 9 or ca 10 Ma for *K. ayeyarwadyensis* while sedimentology and stable isotopes analyses suggest that this hominoid inhabited an evergreen forest in a flood plain. The discovery of *Khoratpithecus ayeyarwadyensis* highlights the increasing diversity of large hominoids in the Miocene of southern Asia. The new species does not change the temporal range of the genus *Khoratpithecus*, but increases its spatial distribution to a large Southeastern Asian region.

Biogeographically, the fauna associated with *K. ayeyarwadyensis* does not display clear affinities to Yunnan faunas, but rather resembles those of the Siwaliks and Thailand which share, for example, *Merycopotamus medioximus* and the suids *Propotamochoerus* and *Hippopotamodon*. Hominoids, in contrast, are different in these three faunal provinces. Thus, the hominoids of Southeast Asia are more regionally diversified than other large mammals. The observed provincialism in Asian Miocene hominoids may reflect distinct plant communities within Asia [59]. But the hypothesis of large rivers and/or topographic highs playing a major role in isolating the distribution ranges of Asian hominoids cannot be discarded.

The extensive exposures of Irrawaddy Formation, combined with their long temporal range (middle Miocene to Pleistocene), suggest a high potential for the understanding of Asian hominoids evolution.

## Materials and Methods

### Study of the fossils

The *Khoratpithecus* material was studied in collections housed at the Department of Archaeology of Mandalay (Ministry of Culture, Myanmar) and the Departement of Mineral Resources of Bangkok (Ministry of Natural Resources and Environment, Thailand). Fossils were measured using digital calipers and, when available, CT-scan data. Comparisons were made by the study of casts (*Ouranopithecus*, *Lufengpithecus*, *Sivapithecus*) and through the literature.

### Nomenclatural Acts

The electronic version of this document does not represent a published work according to the International Code of Zoological Nomenclature (ICZN), and hence the nomenclatural acts contained in the electronic version are not available under that Code from the electronic edition. Therefore, a separate edition of this document was produced by a method that assures numerous identical and durable copies, and those copies were simultaneously obtainable (from the publication date noted on the first page of this article) for the purpose of providing a public and permanent scientific record, in accordance with Article 8.1 of the Code. The separate print-only edition is available on request from PLoS by sending a request to PLoS ONE, 1160 Battery Street, Suite 100, San Francisco, CA 94111, USA along with a check for $10 (to cover printing and postage) payable to “Public Library of Science”.

In addition, this published work and the nomenclatural acts it contains have been registered in ZooBank, the proposed online registration system for the ICZN. The ZooBank LSIDs (Life Science Identifiers) can be resolved and the associated information viewed through any standard web browser by appending the LSID to the prefix “http://zoobank.org/”. The LSID for this publication is: urn:lsid:zoobank.org:pub:F7D261AF-6B13-4029-A51A-32239E579A7C.
